# Ovarian Cystadenofibroma: An Overview

**DOI:** 10.7759/cureus.87614

**Published:** 2025-07-09

**Authors:** Vinodini V, Sunita Samal

**Affiliations:** 1 Obstetrics and Gynaecology, Apollo Hospitals, Chennai, IND

**Keywords:** benign tumour, incidence, laterality, ovarian cystadenofibroma, torsion

## Abstract

Background

An uncommon benign ovarian tumour with both cystic and solid features has been noted with increasing frequency at our institution. Though often asymptomatic, it can occasionally present with abdominal symptoms or menstrual irregularities. This observation prompted an investigation into the clinical and epidemiological patterns of these cases.

Methods

This retrospective observational study included 96 cases of ovarian cystadenofibroma operated on between January 2023 and November 2023. Demographic data, clinical presentation, and radiological, intraoperative, and histopathological findings were assessed. Statistical significance was set at p < 0.05.

Results

Among 96 cases, serous (n = 42, 44%), mucinous (n = 36, 38%), and mixed (n = 18, 19%) subtypes were identified. Statistically significant associations were found between histological subtype and dyslipidaemia (P = 0.0003), heavy menstrual bleeding (P = 0.01), dysmenorrhea (P = 0.03), infertility (P = 0.001), tumour size (P = 0.0001), laterality (P = 0.0001), and torsion (P = 0.03). Mixed tumours in our cohort were larger and more prone to torsion, while serous tumours more frequently showed bilateral involvement. CA-125 levels and IOTA patterns did not show a significant correlation.

Conclusion

A higher-than-expected number of cystadenofibroma cases were observed in our institution. Although benign, these tumours can present with features that mimic malignancy, potentially leading to aggressive surgical management. Early identification through clinical and radiological correlation in our setting may help reduce overtreatment and guide appropriate surgical planning.

## Introduction

Ovarian cystadenofibroma, a type of epithelial ovarian tumour, is a relatively rare benign tumour that contains both epithelial and fibrous stromal components arising from germinal lining and ovarian stroma, seen in women aged >15-65 years [1,2]. It accounts for approximately 1.7% of benign ovarian tumours [3]. In the ovary, it typically manifests as a combination of solid and cystic tumours. The tumour’s solid, semi-solid, or liquid components are determined by the proportions of the stromal and epithelial ingredients as well as the secretory activity of the epithelial components. There are no known risk factors for ovarian cystadenofibroma. However, women who were on hormone replacement treatment and were overweight or menopausal are more vulnerable [4]. Although there may be vaginal bleeding and abdominal pain, it is frequently seen as an asymptomatic incidental tumour. Cystadenofibromas often appear as a single mass inside the ovary, while they can also occasionally appear as several masses inside a single ovary. Bilateral ovaries may be affected in rare cases [5].

The fibrous component of this tumour frequently gives it a specific/characteristic MRI appearance that may help distinguish it from malignant ovarian tumours, even though its regular imaging findings may match those of a malignant neoplasm [6]. The risk of ovarian torsion increases with the size of the ovarian tumour. Thorough sonographic examination and optimization are essential for determining the risk of malignancy and the likelihood of torsion. Tumour markers may not be useful in determining whether ovarian cancers are benign or malignant. Carbohydrate antigen 125 (CA-125) is the most useful tumour marker for detecting ovarian cancer.

Patients diagnosed with these tumours frequently require surgical intervention. These tumours have an excellent prognosis with proper surgical management. However, ovarian cystadenofibroma is an uncommon form of ovarian cancer, and a considerable number of cases have been diagnosed at our institution. Therefore, we aim to investigate the epidemiological, clinical-radiological, and pathological characteristics of these tumours.

## Materials and methods

The study aims to determine the incidence of ovarian cystadenofibroma to analyse the epidemiological, clinico-radiological, and pathological characteristics of these tumours and to determine the correlation between ovarian cystadenofibroma and the various factors associated with it. This is a hospital-based retrospective study undertaken in the department of obstetrics and gynaecology at our institution from January 2023 to November 2023. Before commencing the study, the ethical committee's approval was obtained. A total of 96 patients of different age groups who were reported as having ovarian cystadenofibroma in histopathological reports at our institution were included in the study. Ovarian tumours of other histopathological findings and malignant ovarian tumours were excluded (123 cases). All patients were evaluated pre-operatively with ultrasound, routine blood investigations, and tumour markers and underwent surgery at our institution. Various factors such as demographic characteristics, mode of presentation, radiological factors, intraoperative findings such as cyst diameter, laterality, torsion, and contents of the cyst, and postoperative histopathological findings were documented. The correlation between ovarian cystadenofibroma and the various factors associated with it was determined.

Statistical analyses

In this study, data were collected retrospectively from the medical records department using an Excel sheet (Microsoft Corporation, Redmond, WA) after getting approval from the ethical committee. Descriptive statistics were presented with frequency (percentage) and mean + SD for the categorical and continuous factors, respectively. The Shapiro-Wilk test was used to determine the normality of the data. The chi-square test was used to find out the association between two independent categorical factors. P < 0.05 was considered statistically significant. The incidence of cystadenofibroma was calculated, and all statistical analyses were carried out using SPSS version 28 (IBM Corp., Armonk, NY).

## Results

In our institution, out of 219 ovarian tumours, 96 cases were diagnosed as cystadenofibroma, with an incidence of 43.8%. The most common age group of presentation was 21-30 years (27, 28.13%), followed by 41-50 years (21, 21.88%), above 50 years (18, 18.75%), 8-20 years (15, 16.00%), and 31-40 years (15, 15.63%). Geographically, the highest prevalence was among patients from Bangladesh (28, 29.17%), followed by West Bengal (26, 27.08%), Andhra Pradesh and Assam (18 each, 18.75%), and Tamil Nadu (6, 6.25%). Regarding parity, the majority of patients were multiparous (31, 32.29%), followed by primiparous (25, 26.07%), unmarried (20, 20.83%), nulligravida (19, 19.79%), and one antenatal case (1, 1.04%). The most common presenting symptom was lower abdominal pain (41, 42.71%), followed by heavy menstrual bleeding (23, 23.96%), dysmenorrhea (12, 12.50%), irregular cycles (8, 8.33%), and incidental diagnosis during routine health check-ups (8, 8.33%); additionally, four (4.17%) cases presented with primary infertility, as mentioned in Table 1.

**Table 1:** Demographic and clinical characteristics.

		Frequency (percentage)
Age (years)	8-20	15 (16.00%)
	21-30	27 (28.13%)
	31-40	15 (15.63%)
	41-50	21 (21.88%)
	50+	18 (18.75%)
Location	Andhra Pradesh	18 (18.75%)
	Assam	18 (18.75%)
	Bangladesh	28 (29.17%)
	Tamil Nadu	6 (6.25%)
	West Bengal	26 (27.08%)
BMI	Underweight	10 (10.42%)
	Normal	11 (11.46%)
	Overweight	35 (36.46%)
	Obese	40 (41.67%)
Parity	Nulligravida	19 (19.79%)
	Primigravida	1 (1.04%)
	Unmarried	20 (20.83%)
	Primiparous	25 (26.04%)
	Multiparous	31 (32.29%)
Mode of presentation	Anxious to conceive	4 (4.17%)
	Dysmenorrhea	12 (12.50%)
	Heavy menstrual bleeding	23 (23.96%)
	Irregular cycles	8 (8.33%)
	Lower abdominal pain	41 (42.71%)
	Regular health checkup	8 (8.33%)

Among 96 cases, 48 (50%) had no identifiable risk factors, while 20 (20.83%) had diabetes, 16 (16.67%) had hypertension, and 12 (12.50%) had dyslipidaemia, as mentioned in Table 2. Additionally, 21 patients (21.88%) were postmenopausal.

**Table 2:** Analysis of risk factors for cystadenofibroma.

Risk factors	Frequency (percentage)
Diabetes	20 (20.83%)
Dyslipidemia	12 (12.50%)
Hypertension	16 (16.67%)
Nil	48 (50.00%)

**Figure 1:**
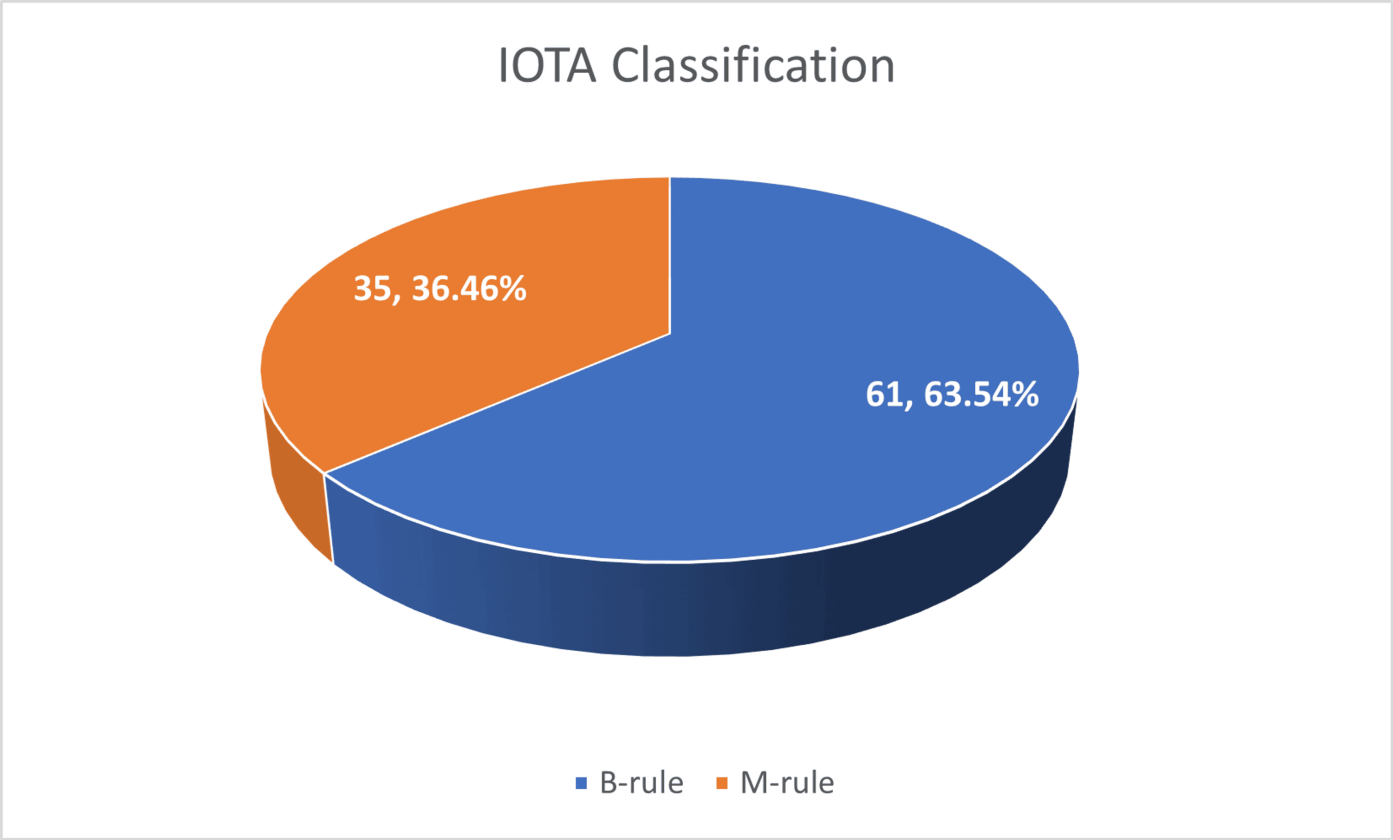
The International Ovarian Tumor Analysis (IOTA) classification.

Based on the International Ovarian Tumor Analysis (IOTA) classification [7], benign tumour traits (B-features) include the following: B1 = unilocular; B2 = presence of solid portions with the greatest solid component < 7 mm in diameter; B3 = presence of sonic shadows; B4 = smooth multilocular tumour with ≤ 10 cm its largest diameter; and B5 = no blood supply. Malignant tumour traits (M-features) include the following: M1 = irregular solid tumour; M2 = presence of ascites; M3 = presence of four or more papillary structures; M4 = irregular multilocular tumour with ≥10 cm maximum diameter; and M5 = very strong blood flow. A total of 61 (63.54%) cases followed B-rules, suggesting benign characteristics, whereas 35 (36.46%) followed M-rules, indicating a suspicion of malignancy, as depicted in Figure 1.

**Figure 2:**
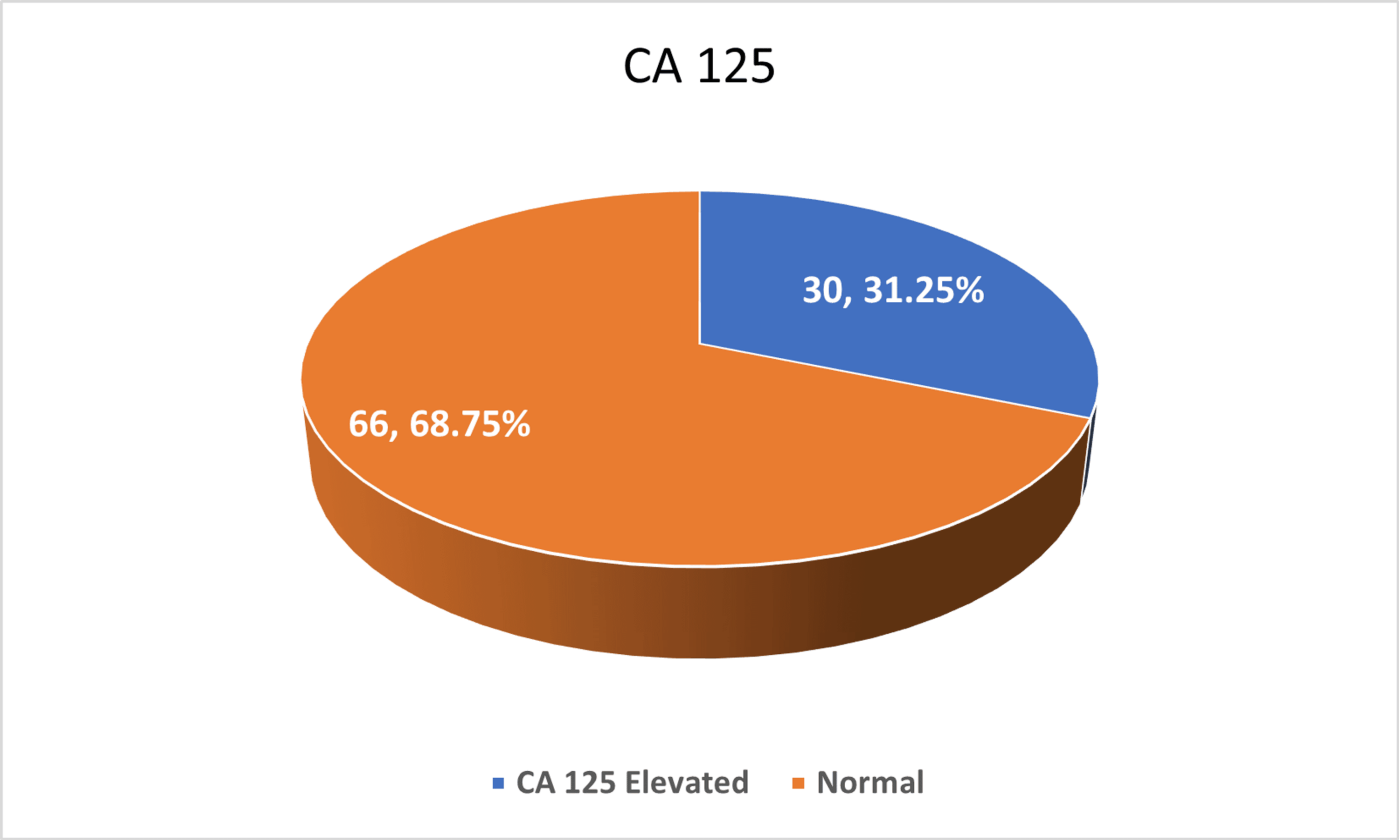
Cancer antigen 125 (CA 125).

According to the National Institute for Health and Care Excellence (NICE) guidelines [8], CA-125 more than or equal to 35 U/ml was considered elevated and less than 35 U/ml was considered normal. CA-125 levels were elevated in only 30 (31.25%), while 66 (68.75%) had normal values, reinforcing the benign nature of most cases, as shown in Figure 2.

Laterality analysis showed 42 (43.75%) cysts were right-sided, 38 (39.58%) were left-sided, and 16 (16.67%) were bilateral. Torsion was observed in 12 (12.50%) cases, primarily in cysts larger than 10 cm, while 84 (87.50%) had no torsion. Regarding cyst contents, 42 (43.75%) were serous, 36 (37.50%) were mucinous, and 18 (18.75%) had mixed or variable contents. The majority of cases (59, 61.46%) involved ovarian cysts, while 37 (38.54%) were para-ovarian cysts. Surgical management included cystectomy in 54 (56.25%), hysterectomy in 36 (37.50%), and oophorectomy in six (6.25%) cases. Laparoscopic surgery was the preferred approach in 91 (94.79%) cases, while open surgery was performed in only five (5.21%), as mentioned in Table 3. In view of the suspicion of malignancy, frozen sections were performed for two patients.

**Table 3:** Intra-operative parameters.

		Frequency (percentage)
Laterality	Left	38 (39.58%)
	Right	42 (43.75%)
	Bilateral	16 (16.67%)
Torsion	Yes	12 (12.50%)
	No	84 (87.50%)
Size	0–5 cm	25 (26.04%)
	5–10 cm	36 (37.50%)
	>10 cm	35 (36.46%)
Contents of the cyst	Serous	42 (43.75%)
	Mucinous	36 (37.50%)
	Variable	18 (18.75%)
Type of cyst	Ovarian	59 (61.46%)
	Para-ovarian	37 (38.54%)
Type of surgery	Cystectomy	54 (56.25%)
	Oophorectomy	6 (6.25%)
	Hysterectomy	36 (37.50%)
Mode of surgery	Laparoscopy	91 (94.79%)
	Open	5 (5.21%)

In this study, we evaluated the correlation between various clinical, demographic, and radiological parameters with different histological subtypes of ovarian cystadenofibroma (serous, mucinous, and mixed types) among 96 patients. Age distribution did not show a statistically significant association with the histological subtype (P = 0.42), although the 21-30 years age group was the most commonly affected (n = 27), followed by 41-50 years (n = 21). Among risk factors, dyslipidaemia showed a statistically significant correlation with the histological subtype (P = 0.0003), with a higher prevalence in serous tumours. Hypertension and diabetes were noted across all types but did not reach statistical significance. Interestingly, 48 out of 96 patients (50%) had no identifiable risk factors.

Regarding clinical presentation, significant associations were observed in several symptom categories. Heavy menstrual bleeding was more frequently associated with mixed-type tumours (P = 0.01), while dysmenorrhea showed a stronger association with serous and mixed subtypes (P = 0.03). Notably, women who presented with infertility-related concerns (“anxious to conceive”) were predominantly found to have mucinous tumours, and this correlation was statistically significant (P = 0.001). Although lower abdominal pain was the most common mode of presentation across all subtypes (n = 41), it did not show a significant association (P = 0.15). Other symptoms, such as irregular menstrual cycles (P = 0.07) and incidental findings during routine check-ups (P = 0.93), were not significantly associated with tumour type.

Radiological evaluation using pattern-based classification (B-rule and M-rule) did not show a significant correlation with histologic subtype (P = 0.24). However, B-rule patterns were more common overall, especially in serous tumours. Tumour marker analysis (CA-125) showed elevated levels in 30 cases, predominantly in mixed-type tumours, but this was not statistically significant (P = 0.28).

Tumour size, however, demonstrated a strong statistical correlation with histological subtype (P = 0.0001). Mixed-type tumours tended to be larger, with 12 out of 19 being more than 10 cm in size, while serous tumours were more frequently in the 5-10 cm range. Laterality also showed a highly significant association (P = 0.0001), with serous tumours more often presenting bilaterally (n = 28) compared to mucinous and mixed types, which were predominantly unilateral. Torsion was present in 12 cases and showed a statistically significant association with tumour subtype (P = 0.03), being more frequent in mixed-type tumours, as shown in Table 4.

**Table 4:** Correlation of various factors in relation to cystadenofibroma.

Factors		Serous (n = 52)	Mucinous (n = 25)	Mixed type (n = 19)	Total count (n = 96)	p-value
Age group (years)	8-20	12	2	1	15	0.4256
	21-30	10	11	6	27	
	31-40	10	3	2	15	
	41-50	11	5	5	21	
	50+	9	4	5	18	
Risk factors	Diabetes	11	5	4	20	0.0003
	Dyslipidemia	9	3	0	12	
	Hypertension	9	3	4	16	
	Nil	23	14	11	48	
Mode of presentation	Anxious to conceive	1	3	-	4	0.0018
	Dysmenorrhea	7	2	3	12	0.0323
	Irregular cycles	6	1	1	8	0.0765
	Lower abdominal pain	18	16	7	41	0.1543
	Heavy menstrual bleeding	14	2	7	23	0.0133
	Regular health check-up	6	1	1	8	0.9322
Radiological features	B-rule	31	17	13	61	0.2436
	M-rule	21	8	6	35	
Tumour marker (CA-125)	Elevated	13	8	9	30	0.2801
	Normal	39	17	10	66	
Size category	0-5 cm	16	6	3	25	0.0001
	5-10 cm	22	10	4	35	
	10+ cm	14	9	12	36	
Laterality	Bilateral	17	7	4	28	0.0001
	Unilateral	35	18	15	68	
Torsion	No	46	23	15	84	0.0312
	Yes	6	2	4	12	

## Discussion

In our study, cystadenofibromas constituted 43.8% of all ovarian tumours (96 out of 219), a notably higher incidence compared to previous reports where these tumours are generally considered rare. For instance, Leelavathi et al. and Singh et al. reported cystadenofibroma as isolated case reports, underscoring the relative scarcity of this tumour type in general practice [1,5]. Our larger dataset allowed for a more robust analysis of demographic and clinical correlations, contributing valuable insights to the limited literature available on this entity.

The most commonly affected age group in our cohort was 21-30 years (27, 28.13%), with a gradual decline in incidence in older age groups. This is in contrast to other studies, such as Fujita et al. and Bencherifi et al. [3,4], where most reported cases involved perimenopausal or postmenopausal women. Notably, our study had a relatively younger population, possibly due to referral patterns and demographic characteristics, as a significant number of cross-border referrals from Bangladesh and the northeastern states of India.

In terms of clinical presentation, lower abdominal pain was the most frequent symptom (41, 42.71%), consistent with findings from Mangla et al. and Krohn et al., who noted intermittent pain often due to torsion or mass effect [9,10]. However, our study went further to statistically associate certain symptoms with histological subtypes; heavy menstrual bleeding and dysmenorrhea were significantly associated with mixed and serous types, respectively. Most of the patients who had heavy menstrual bleeding were found to have associated uterine pathologies. This level of symptom-subtype analysis has been underexplored in prior case-based literature, offering new perspectives for symptom-guided differential diagnosis.

Biochemical markers such as CA-125 were elevated in only 30 (31.25%) of our cases, predominantly in mixed tumours, a finding comparable with Kim et al. who cautioned against over-reliance on CA-125 in complex benign tumours [11]. This underlines the potential of cystadenofibroma to mimic malignancy both clinically and radiologically, leading to overtreatment in some cases.

The incidence of torsion in our study was 12 (12.5%), aligning with reports from Ihssan et al., who documented a case of torsion involving a large haemorrhagic cystadenofibroma [12]. The association of torsion with larger tumour size in our cohort further strengthens the clinical importance of early imaging and intervention in adnexal masses larger than 10 cm, a finding also supported by Bo et al., who emphasised the role of MRI in preoperative assessment of complex fibrous ovarian lesions [13].

From a radiological perspective, 61 (63.54%) of our cases fulfilled the IOTA B-rules, indicating benign features, and only 35 (36.46%) met the M-rule criteria, even though histopathological examination shows benign pathology in our study, echoing the benign imaging profile described by Wasnik et al. [2]. However, we found no statistically significant correlation between the IOTA classification and histological subtype, which suggests that while useful for benign-malignant differentiation, the IOTA criteria may not reliably predict subtypes.

Significantly, our study identified dyslipidaemia as a novel and statistically significant risk factor associated with serous cystadenofibromas (P = 0.0003), a finding not previously highlighted in the literature. This warrants further exploration in future prospective studies to determine if lipid metabolism plays a role in the pathogenesis of these tumours.

In summary, our findings correlate well with earlier case-based reports but expand the knowledge base through statistical validation of symptom-subtype associations and identification of novel risk factors. This study underscores the importance of integrating clinical, radiological, and biochemical profiles in the diagnosis and tailored management of ovarian cystadenofibromas.

This study has certain limitations that should be acknowledged. As a retrospective study, it is dependent on the accuracy and completeness of existing medical records, and the absence of long-term follow-up data limits conclusions regarding recurrence or prognosis. Although the sample size is substantial for a rare tumour type, further validation in larger, multicentre cohorts would strengthen the findings. Additionally, while CA-125 was assessed, the inclusion of a broader panel of tumour markers and consistent use of advanced imaging such as MRI could enhance diagnostic accuracy. The correlation of CA-125 levels with menopausal status could not be assessed due to the unavailability of consistent menopausal data across participants. This limits the ability to interpret CA-125 elevation in the context of physiological menopausal variations. Despite these constraints, the study contributes significantly to the limited literature on cystadenofibroma and underscores important patterns that warrant further prospective investigation.

## Conclusions

The present study observed a higher number of ovarian cystadenofibroma cases (96, 43.8%) than typically expected, suggesting possible regional or institutional variations. Although cystadenofibroma is a benign tumour, its close resemblance to malignant ovarian masses often leads to aggressive surgical management and potential complications. With appropriate surgical intervention, however, outcomes remain excellent. This study underscores the importance of recognizing its clinical and imaging features to aid in accurate diagnosis and avoid unnecessary extensive procedures. Early identification can help reduce overtreatment, improve surgical decision-making, and optimize patient outcomes. These findings also point toward distinctive epidemiological patterns that merit further investigation.
